# Sex-specific inbreeding depression of the life-history traits in *Frankliniella occidentalis*

**DOI:** 10.1093/jisesa/ieag121

**Published:** 2026-11-06

**Authors:** Wondimagegn Atilaw Woldemelak

**Affiliations:** Department of Horticulture, Debre Berhan University, Debre Berhan, Ethiopia

**Keywords:** haplodiploidy, inbreeding depression, life table, fluctuating asymmetry, Thysanoptera, pest management

## Abstract

Inbreeding depression decreases the fitness of progeny resulting from mating between related individuals. The sex-specific response to inbreeding remains unknown in haplodiploid organisms, which have haploid males and diploid females. This research examines the consequences of inbreeding using 2 different inbreeding protocols: full-sib (brother–sister, BS) and maternal backcross (mother–son, MS) on life history, demographic variables, growth in population size, and morphological stability in *Frankliniella occidentalis*. The experiment was tested for 3 generations: 1 outbred (F_1_) and 2 inbred (F_2_ and F_3_) for both the BS and MS inbreeding regimes. Sibling mating reduced lifetime reproductive productivity from 101.00 to 60.72 (39.9% reduction); maternal backcrossing led to an even greater reduction from 100.20 to 39.10 (61.3% reduction) by F_3_ generation. Second-clutch egg number declined from 35.42 (control) to 17.85 (BS F_3_) and 6.45 (MS F_3_), representing 49.6% and 81.8% reductions, respectively. Net reproductive rate (*R*_0_) was reduced from 48.52 to 14.15 (sibling) and to catastrophic levels of 4.85 (backcross; 90.0% reduction). Intrinsic rate of increase (*r*) was sharply reduced from 0.185 d^−^^1^ to 0.088 d^−^^1^ (sibling) and 0.024 d^−^^1^ (backcross; 87.0% reduction), while the finite rate of increase (*λ*) declined from 1.203 to 1.092 and 1.024, respectively. Mean generation time (*T*) increased from 21.05 to 30.12 d (sibling) and 34.60 d (backcross; 64.3% increase). In the case of maternal backcrossing, there was no purging of deleterious alleles in males due to the haploid phase, since their survival rate dropped drastically to 41.4% and malformation levels reached 32.2% overall, with nearly equal distribution between sexes (16.2% in females and 16.0% in males).

## Introduction

The phenomenon of inbreeding depression, which refers to a decrease in fitness in the offspring of closely related parents, is mainly associated with the higher activity of harmful recessive alleles and the lack of heterozygote advantage at overdominance loci (Charlesworth and Willis 2009, Hedrick and Garcia-Dorado 2016). For diploid organisms, the effects of inbreeding on fitness are usually similar for both sexes, unless there are sex-linked genes or sexual antagonism (Hedrick 2012, Szulkin et al. 2010). However, in haplodiploids, the pattern of inbreeding effects is very different. Males develop from unfertilized haploid eggs through arrhenotokous reproduction, where unfertilized eggs develop into males and fertilized eggs develop into females. Because deleterious recessive alleles are subject to natural selection when present in a single copy in haploid males, this ought to result in them being purged more effectively than they would be in diploid females, which may carry the alleles in a heterozygous condition (Hedrick 1994, Glémin et al. 2019). Because of this, haplodiploids were traditionally expected to suffer less inbreeding depression, especially for hemizygous traits (Pannebakker et al. 2011, Barrett and Charlesworth 1991).

Although the above-mentioned theoretical predictions exist, there is an increasing number of empirical studies showing significant inbreeding depression in various haplodiploids with respect to important fitness traits like body size, developmental rate, lifespan, and reproductive success (Leung et al. 2025). The aforementioned contradiction indicates that inbreeding purge is not an unconditionally effective process, being influenced by several aspects. In particular, the nature of the mating system, the sex specificity and dominance of harmful alleles, the effective population size, and the rate of inbreeding progression in each generation should be taken into account (Willoughby et al. 2019). Also, various inbreeding schemes, such as mating between full siblings as compared to backcrossing to mothers from sons, may have different impacts due to differences in the increase in homozygosity and the transfer of sex-linked or biased genes (Wang 1996). One-generational experiments cannot reflect the accumulative impact of successive inbreeding, while multi-generational studies are vital for revealing whether haplodiploids provide genetic resistance to inbreeding or postpone its negative impact manifestation.

*Frankliniella occidentalis* (Pergande, 1895) (Thysanoptera: Thripidae), commonly referred to as the western flower thrips, can be considered an excellent study system in understanding these processes. This is due to the fact that the western flower thrips is a worldwide invasive insect pest whose activities lead to great economic losses to agricultural products by causing direct feeding, oviposition damage, as well as transmitting some destructive plant viruses like Tomato spotted wilt virus (TSWV) (Riley et al. 2011, Rotenberg et al. 2015). The western flower thrips breeds through arrhenotoky (Mound and Kibby 1998, Moritz et al. 2004). Intensively managed agricultural systems, in which there is usually a history of bottlenecks, high fidelity to local sites, and spatial clustering, are an environment that fosters repeated mating between siblings and/or between mothers and sons (Kirk and Terry 2003, Reitz et al. 2020). Although much work has been done on the ecology, behavior, and resistance to insecticides in this species (Demirozer et al. 2012), little attention has been paid to how the reproductive fitness of individuals from multiple generations of these natural instances of inbreeding varies with regard to sexual life histories and other demographically related traits.

To fill this knowledge gap, the objectives of the current study include (i) estimating the degree and trend of inbreeding depression due to sexual reproduction in *F. occidentalis* within 3 successive generations for 2 types of inbreeding systems: full-sib mating and backcross mating; (ii) comparing the fitness costs associated with the 2 forms of inbreeding with respect to the 2-sex life table analysis, population growth characteristics such as net reproductive rate and intrinsic rate of natural increase, as well as other life history traits in both males and females; and (iii) evaluating the impact of successive inbreeding on developmental stability, through the occurrence of morphological anomalies and fluctuating asymmetries.

## Materials and Methods

### Stock Colony

The experiment was initiated using a cohort of 30 adult female *F. occidentalis* randomly selected from a long-established outbred stock colony maintained on green bean pods (*Phaseolus vulgaris* L.) at 25 ± 1 °C, 60 ± 5% RH, and a 12:12 h (L: D) photoperiod. In order to maintain consistency in the maternal environment, each chosen female (F_0_) was carefully moved to a sterile 2 ml microcentrifuge tube that was used as a unique rearing arena. In each tube, a freshly cut green bean leaf disc that had a size of about 15 mm in diameter was offered as a single source of food and oviposition substrate. The founding females were kept in a specific incubator with a standard temperature of 23 ± 1 °C, where the relative humidity was also standardized at 60 ± 5%.

### Experimental Design and Inbreeding Regimes

Experimental populations were established for two inbreeding regimes, brother–sister (BS) and mother-son (MS), beginning with the F_0_ parental generation. For all treatments, the F_1_ generation was outbred and produced by mating unrelated F_0_ individuals. In the BS regime, 1 BS pair from F_1_ was randomly chosen and mated to produce BS F_2_ (first inbred generation), and 1 brother-sister pair from BS F_2_ was randomly chosen and mated to produce BS F_3_ (second inbred generation). In the MS regime, each F_0_ mother was mated to one of its own F_1_ sons to produce MS F_2_ (first MS inbred generation), and each MS F_2_ mother was mated to one of its own sons to produce MS F_3_ (second mother-son inbred generation). Thus, both regimes involved 1 outbred generation (F_1_) and 2 successive inbred generations (F_2_ and F_3_). The Control consisted of outbred F_1_, F_2_, and F_3_ generations maintained in parallel under identical conditions. For each treatment (BS, MS, Control) and generation, 20 independent replicate lines were established, each founded by a single mated female. Lines were propagated separately to maintain independent evolutionary trajectories. To minimize genetic drift, at least 30 individuals were maintained at each generation before selecting the next generation’s parents.

#### Sibling (BS) inbreeding lines

To establish the BS lines, late-stage pupae were selected from the outbred stock colony and reared to adulthood to obtain virgin females and virgin males. Each virgin female was placed individually in a rearing tube with a virgin male from a different line to produce the F_1_ generation (outbred, since parents were unrelated). From this F_1_ generation, 1 BS pair was randomly chosen and mated to produce the F_2_ generation (1st sibling mating, first inbred generation). From the F_2_ generation, 1 BS pair was randomly chosen and mated to produce the F_3_ generation (second sibling mating, second inbred generation). Thus, the BS treatment involved 1 outbred generation (F_1_) and 2 successive generations of sibling inbreeding: F_2_ (first sibling mating) and F_3_ (second sibling mating). F_1_ was outbred and served as the starting generation for the inbreeding protocol.

#### MS backcross lines

The MS inbreeding protocol was designed to produce 1 outbred generation (F_1_) and 2 successive generations of MS inbreeding (MS F_2_ and MS F_3_). The protocol began with virgin F_0_ females obtained from late-stage pupae of the outbred stock colony. Each virgin F_0_ female was placed individually in a rearing tube with an unrelated F_0_ male from the outbred stock colony to produce the F_1_ generation (outbred, since parents were unrelated). Because *F. occidentalis* reproduces through arrhenotoky, unmated females produce only haploid male offspring. These F_1_ sons were collected as late pupae and reared to adulthood to ensure virginity. Once the F_1_ sons reached adulthood, each F_0_ mother was mated to its own F_1_ son. This first MS mating produced the MS F_2_ generation, which represents the first generation of MS inbreeding. The MS F_2_ females were then isolated as virgins and allowed to produce their own sons. Each MS F_2_ mother was subsequently mated to its own son. This second MS mating produced the MS F_3_ generation, which represents the second generation of MS inbreeding. Thus, the MS treatment involved 1 outbred generation (F_1_) and 2 successive generations of MS inbreeding: MS F_2_ (first MS mating) and MS F_3_ (second MS mating).

### Cold-Storage Protocol

To ensure that the mother survived until its sons reached reproductive maturity in the backcross mating treatment, and to standardize the maternal thermal environment across all MS generations, the same cold-storage protocol was applied to every female that produced for MS inbreeding at different generations (MS F_1_, MS F_2_, and MS F_3_). After mating, each female was moved to a new rearing tube within 1 h of oviposition and kept at 15 ± 1 °C under a 12:12 h light regime for 14–16 d. It was then acclimated at 23 °C for 2 h before oviposition. This protocol was applied to: F_0_ females before production of MS F_1_, F_0_ females before production of MS F_2_, after mating with a son produced by the same female, MS F_2_ females before production of MS F_3_, after mating with a son produced by the same female. Thus, all MS generations were produced by cold-stored females. The Control and BS regimes were not cold-stored, and this difference is considered explicitly in the interpretation of between-regime comparisons.

### Sham-Control Experiment

To address potential confounding effects of the cold-storage protocol, a separate sham-control experiment was conducted. Twenty outbred females from the same stock colony were subjected to the identical temperature manipulation protocol (15 ± 1 °C for 14–16 d, followed by 2 h acclimation at 23 °C) used in the backcross treatment. These females were then mated to unrelated males at 23 °C and their reproductive parameters were assessed (Table 1).

**Table 1.** Results of sham-control experiment

Parameter	Control (no cold)	Cold-exposed (15 °C, 14–16 d)	% Change	*P*-value
**Lifetime egg number**	101.00 ± 4.87 (*N* = 20 females)	96.80 ± 5.12 (*N* = 20 females)	−4.2%	0.342
**Hatching success (%)**	83.95 ± 3.97 (*N* = 1,530 eggs)	80.15 ± 4.20 (*N* = 1,460 eggs)	−4.5%	0.401
**Female survival (%)**	84.2 ± 3.5 (*N* = 20 females)	79.8 ± 4.1 (*N* = 20 females)	−5.2%	0.286
**Intrinsic rate (*r*)**	0.185 ± 0.011 (*N* = 20 females)	0.178 ± 0.012 (*N* = 20 females)	−3.8%	0.318

### Mating Procedures and Offspring Synchronization

Each MS pair as well as each sibling pair was kept in a clean 5 ml microcentrifuge tube containing a freshly prepared green bean leaf disk as a source of nutrition and oviposition surface. These tubes were provided ventilation using a fine mesh with a perforated top, and they were kept at 23 ± 1 °C, 60 ± 5% relative humidity, and a 12:12 h photoperiod. They were left to mate for 48 h after which the males were gently aspirated off so that they could not have any further mating or any kind of male-induced stress.

*F. occidentalis*, sex determination is arrhenotokous, where fertilized (2*n*) eggs form females and unfertilized (*n*) eggs form males (Lewis 1973). Mating was regarded as successful if a female formed a mixed-sex offspring including both sexes. On the other hand, mating was regarded as unsuccessful if a female only gave birth to male offspring. To ensure that the targeted replication numbers for each treatment group in each generation of 20 replicates were maintained, replacement virgin females of the same generation were kept separately and replaced only when their exclusion from the study was confirmed. In order to avoid any bias caused by age, all the progeny were synchronized based on generation, where each adult generation was collected daily and those hatching within the same 24-h period were pooled together into age groups (±1 d) following the methodology of Chi (1988). This synchronization was applied consistently across all treatments and generations.

### Assessment of Reproductive and Life-History Traits

For each mated female, the number of eggs produced was separated into discrete temporal clutches. The first clutch information was obtained from the number of eggs laid within the first 5 d following mating (days 1–5). The second clutch information was obtained from the number of eggs laid between days 6 and 10 after mating. The leaf discs were changed at an interval of 48 h; the changed leaf discs were examined using a stereomicroscope to determine the number of eggs produced as well as the number of hatched eggs. The number of eggs for each clutch was obtained by adding the total number of eggs laid per leaf disc within a specific clutch period.

The number of newly hatched nymphs was expressed as the actual count of hatched larvae per day of monitoring for each clutch period. Lifetime egg number is the total number of eggs laid by a single female from the start of its oviposition until death, whereas lifetime newly hatched nymphs is the total number of viable hatched larvae from its whole life span. The probability of laying another clutch was estimated based on the proportion of females that laid second clutches out of the total number of females that had laid first clutches, expressed as a percentage. The reproductive investment into second clutches was measured as the ratio of reproductive effort (number of eggs/nymphs) in the second clutch relative to the first clutch, estimated as the ratio of second clutch production to the first one, expressed as a percentage. All mated females were included in the analysis, regardless of whether they produced eggs. This intention-to-treat approach follows standard life table methodology (Chi 1988).

### Sex-Specific Survival and Longevity Assessment

After the 48 h of mating, removal of the males, and daily recording of survival rates of the females at 09:00 h every day. Adult lifespan of the female was the period between adult eclosion and death. Survival rate of females per generation was calculated as the percentage of females that survived the entire reproductive period in relation to the initial number of females. The survival rate of males was obtained through monitoring daily survival of adult progeny males from eclosion to death. The survival rate of males was determined as the percentage of males surviving to reproductive maturity (eclosion as adults) and throughout the observation period. In mother-to-son crosses, the assurance of the virginity of males was done through the isolation of late-stage pupae in individual vials to ensure that only the required backcross matings took place.

### Morphological Assessment

Morphological inspection was carried out on all the offspring at adult emergence using a stereomicroscope (Leica MZ16) with up to 100× magnification according to Palmer and Strobeck (1986). Malformations of phenotype were measured using binary (present/absent) data. Some of the malformations noted include wing malformation (insects with crumpled wings, smaller or asymmetrical wings), tergite malformation (insects with deformed tergites), and antennal fusion (insects with fusion or deformation of their antennal segments). Malformation rates were calculated based on the total number of adult progeny examined. The malformation frequency was obtained by dividing the number of insects having any morphological malformations by the total number of insects, then multiplying by 100, calculated separately for each treatment, sex, and generation. The denominator for malformation rates was the total number of individual adult progeny examined pooled across all 20 replicate lines (*N* = 800 per treatment × generation combination). The estimation of fluctuating asymmetry (FA) was carried out following the method developed by Palmer and Strobeck (1986). FA was estimated as the absolute value of the difference between right and left measurements for each individual organism: FA = |Right measurement − Left measurement| (Palmer and Strobeck 1986).

### Data Processing and Life Table Analysis

Raw biological data-including developmental times, stage-specific survival, adult longevity, daily oviposition, daily hatching, adult emergence, and sex of each individual were analyzed according to age-stage, 2-sex life table theory using TWOSEX-MSChart (Chi 2024), with data for both male and female individuals included in the underlying age-stage structure.

The life-table workflow and parameter estimation were conducted sequentially starting with individual records, where each subject was followed from birth to death or censoring with age, stage, sex, survival, and reproductive output recorded daily. The age-stage-specific survival rate (*S_xj_*), calculated as Sxj=nxjn0, (where *n_xj_* is the number of individuals alive at age *x* and stage *j*, and *n*_0_ is the initial cohort size), represented the probability that a newborn individual survives to age *x* and stage *j*. This calculation incorporated both male and female individuals, and non-emerged or dead individuals were retained as mortality in *S_xj_* rather than excluded. Next, the overall age-specific survival rate was calculated as $lx=\sum_{j=1}^{k}\mathrm{Sxj}$, where *k* is the total number of stages, ensuring that male and female developmental times and stage-specific survival were fully integrated into *S_xj_* and *l_x_*. For each treatment *x* generation *x* age *x*, the daily progeny sex ratio was calculated directly from observed adult emergence as pf,x=Nf,x(Nf,x+Nm,x), where *N_f,x_* and *N_m,x_* represent the numbers of female and male adults emerging on day *x* from synchronized cohorts. Because TWOSEX-MSChart requires age-specific fecundity *m_x_* to be defined as female offspring per female at age *x*, total daily offspring production was converted to female offspring using the observed daily sex ratio *pf,x* alongside stage-specific hatching or emergence success where appropriate. Age-specific fecundity was subsequently calculated as $mx=(\sum_{j=1}^{k}\mathrm{Sxjfxj})/\sum_{j=1}^{k}\mathrm{Sxj}$, where *fxj* is female offspring produced per female at age x and stage *j*, set to *fxj *= 0 for male and immature stages.

Finally, key population growth parameters were derived using standard formulas: net reproductive rate *R*_0_=$\sum_{X=0}^{\infty }\mathrm{lxmx}$, intrinsic rate of increase *r* via $\sum_{x=0}^{\infty }e^{-r(x+1)}l\mathrm{xmx}=1$, infinite rate of increase *λ = e^r^*, and mean generation time *T* = (ln *R*_0_)/*r*. Standard errors and confidence intervals for all population parameters were estimated using 100,000 bootstrap replicates. Notably, while these population parameters (*R*_0_, *r*, *λ*, and *T*) were calculated based on age-stage, two-sex life table theory, age-specific fecundity (*mx*) was defined strictly as female offspring per female (*mx*, female) to maintain demographic precision; thus, *R*_0_ and *r* represent female-centric metrics, even though both male and female developmental and survival parameters (*sxj*, *lx*) were fully integrated into the underlying age-stage structure.

### Statistical Analysis

All analyses were conducted in R v4.3.1 (R Core Team 2023). Generalized linear mixed models (GLMMs) were used with replicate line ID as a random intercept to account for non-independence among measurements from the same inbred line across generations. Inbreeding type (BS, MS, Control), generation (F_1_–F_3_), sex (female, male), and their interactions were fixed effects, and Line_ID (20 replicate lines per treatment) was a random intercept. Interactions were included to assess sex-specific and generation-dependent effects. Error distributions were specified according to data type: binomial (logit) for proportional data (hatching success, survival, malformation, second-clutch likelihood); Poisson (log) for counts (egg numbers, nymph counts), except overdispersed nymph counts, which used negative binomial; and Gaussian (identity) for continuous data (FA, nymph weight). Model diagnostics included overdispersion assessment, zero-inflation testing using DHARMA (Hartig 2022), and model fit evaluation via AIC and residual plots. No significant zero-inflation was detected (*P* > 0.10 for all DHARMA tests). Offspring sex ratios were analyzed using binomial GLMMs with line-level random intercepts. For FA, comparisons were made between generations within each inbreeding treatment (BS and MS) separately. Parametric *F*-tests were used for omnibus comparisons following Palmer and Strobeck (1986), and *t*-tests were used for specific 2-group comparisons. *Post hoc* comparisons among treatment-generation combinations used Tukey’s HSD to control the family-wise error rate. All values are presented as means ± standard errors (SE). Statistical significance was set at *α* = 0.05.

## Results

### Sham-Control Experiment

The sham-control experiment revealed that the cold-storage protocol caused only small and non-significant reductions across all measured fitness parameters (Table 1). Lifetime egg production declined by 4.2%, from 101.00 ± 4.87 eggs per female in the control group to 96.80 ± 5.12 eggs per female in the cold-exposed group; this reduction was not statistically significant (*P* = 0.342). Similarly, hatching success decreased by 4.5%, from 83.95 ± 3.97% to 80.15 ± 4.20%, with the difference being non-significant (*P* = 0.401). Female survival showed a 5.2% reduction, declining from 84.2 ± 3.5% in controls to 79.8 ± 4.1% in cold-exposed females, which was also not statistically significant (*P* = 0.286). Finally, the intrinsic rate of increase (*r*) declined by 3.8%, from 0.185 ± 0.011 d^−^^1^ in the control group to 0.178 ± 0.012 d^−^^1^ in the cold-exposed group, and this difference was likewise non-significant (*P* = 0.318).

### First-Clutch Reproductive Parameters

#### First-Clutch Egg Number

The number of eggs laid during the first 5 d post-mating declined progressively across generations within each inbreeding regime (Table 2). In outbred controls, first-clutch egg number was 66.52. Within the BS regime, first-clutch egg numbers declined significantly from 64.85 (F_1_) to 55.41 (F_2_) and 48.12 (F_3_) (*F*(2,57) = 10.73, *P* < 0.001), with BS F_3_ representing a 27.7% reduction compared with controls. Within the MS regime, first-clutch egg numbers declined from 65.20 (F_1_) to 46.15 (F_2_) and 34.20 (F_3_) (*F*(2,57) = 16.24, *P* < 0.001), with MS F_3_ representing a 48.6% reduction compared with controls.

**Table 2.** First-clutch reproductive parameters across generations (mean ± SE)

Parameter	Control	BS F_1_	BS F_2_	BS F_3_	MS F_1_	MS F_2_	MS F_3_
**Egg number**	66.52 ± 2.23^a^ (*N* = 20)	64.85 ± 2.10^a^ (*N* = 20)	55.41 ± 1.90^b^ (*N* = 20)	48.12 ± 2.11^c^ (*N* = 20)	65.20 ± 2.15^a^ (*N* = 20)	46.15 ± 1.68^c^ (*N* = 20)	34.20 ± 1.95^d^ (*N* = 20)
**Hatching success (%)**	83.95 ± 3.20^a^ (*N* = 1,530 eggs)	76.40 ± 3.35^a^ (*N* = 1,420 eggs)	65.78 ± 3.20^b^ (*N* = 1,250 eggs)	58.40 ± 3.10^b^ ^c^ (*N* = 1,080 eggs)	82.10 ± 3.25^a^ (*N* = 1,480 eggs)	62.55 ± 3.30^b^ ^c^ (*N* = 980 eggs)	41.15 ± 2.95^d^ (*N* = 720 eggs)
**Newly hatched nymphs**	55.35 ± 3.85^a^ (*N* = 20)	49.20 ± 3.60^a^ (*N* = 20)	36.45 ± 3.20^b^ (*N* = 20)	28.10 ± 2.95^b^ ^c^ (*N* = 20)	53.80 ± 3.75^a^ (*N* = 20)	28.87 ± 2.80^b^ ^c^ (*N* = 20)	14.05 ± 2.10^d^ (*N* = 20)
**Mean nymph weight (mg)**	1.45 ± 0.03^a^ (*N* = 160)	1.46 ± 0.04^a^ (*N* = 180)	1.40 ± 0.04^a^ ^b^ (*N* = 120)	1.31 ± 0.04^b^ ^c^ (*N* = 85)	1.44 ± 0.03^a^ (*N* = 160)	1.32 ± 0.04^b^ ^c^ (*N* = 95)	1.18 ± 0.05^c^ (*N* = 40)

Note: Within each inbreeding treatment (BS or MS), means followed by the same letter (a, b, c, ab, bc) are not significantly different (*P* > 0.05); means followed by different letters are significantly different (*P* < 0.05). Letters compare generations within the same treatment only.

#### First-Clutch Hatching Success Rate

Hatching success in the first clutch followed a similar trajectory of progressive decline within each inbreeding regime. Outbred controls exhibited 83.95% hatching success (Table 2). Within the BS regime, hatching success declined significantly from 76.40% (F_1_) to 65.78% (F_2_) and 58.40% (F_3_) (*F*(2,57) = 9.45, *P* < 0.001). Within the MS regime, hatching success declined from 82.10% (F_1_) to 62.55% (F_2_) and 41.15% (F_3_) (*F*(2,57) = 15.58, *P* < 0.001), with MS F_3_ representing a 51.0% reduction compared with controls.

#### First-Clutch Newly Hatched Nymph Count

The number of newly hatched nymphs from the first clutch fell significantly across generations within each inbreeding regime (Table 2). Outbred controls produced 55.35 nymphs. Within the BS regime, nymph counts declined from 49.20 (F_1_) to 36.45 (F_2_) and 28.10 (F_3_) (F(2,57) = 13.15, *P* < 0.001), with BS F_3_ representing a 49.2% reduction compared with controls. Within the MS regime, nymph counts declined from 53.80 (F_1_) to 28.87 (F_2_) and 14.05 (F_3_) (*F*(2,57) = 20.91, *P* < 0.001), with MS F_3_ representing a 74.6% reduction compared with controls.

#### First-Clutch Mean Nymph Weight

Mean nymph weight from the first clutch showed a transient increase in BS F_1_ (1.46 mg) compared with controls (1.45 mg), before declining in subsequent generations within the BS regime. Within the BS regime, mean nymph weight declined significantly from 1.46 mg (F_1_) to 1.40 mg (F_2_) and 1.31 mg (F_3_) (*F*(2,57) = 4.82, *P* = 0.011). Within the MS regime, mean nymph weight declined from 1.44 mg (F_1_) to 1.32 mg (F_2_) and 1.18 mg (F_3_) (*F*(2,57) = 7.14, *P* < 0.001), with MS F_3_ representing an 18.6% reduction compared with controls (Table 2).

### Second-Clutch Reproductive Parameters (Days 6–10)

#### Second-Clutch Egg Number

The number of eggs laid during the second clutch declined progressively across generations within each inbreeding regime (Table 3). In outbred controls, second-clutch egg number was 35.42 ± 2.18 (*N* = 19). Within the BS regime, second-clutch egg numbers declined significantly from 33.50 ± 2.10 (F_1_) to 24.67 ± 1.89 (F_2_) and 17.85 ± 1.62 (F_3_) (*F*(2,57) = 14.08, *P* < 0.001), with BS F_3_ representing a 49.6% reduction compared with controls. Within the MS regime, second-clutch egg numbers declined from 34.20 ± 2.15 (F_1_) to 18.23 ± 1.75 (F_2_) and 6.45 ± 1.02 (F_3_) (*F*(2,57) = 24.89, *P* < 0.001), with MS F_3_ representing an 81.8% reduction compared with controls.

**Table 3.** Second-clutch reproductive parameters across generations (mean ± SE)

Parameter	Control	BS F_1_	BS F_2_	BS F_3_	MS F_1_	MS F_2_	MS F_3_
**Egg number**	35.42 ± 2.18^a^ (*N* = 19)	33.50 ± 2.10^a^ (*N* = 20)	24.67 ± 1.89^b^ (*N* = 13)	17.85 ± 1.62^c^ (*N* = 9)	34.20 ± 2.15^a^ (*N* = 19)	18.23 ± 1.75^c^ (*N* = 10)	6.45 ± 1.02^d^ (*N* = 4)
**Hatching success (%)**	79.35 ± 3.12^a^ (*N* = 680 eggs)	64.20 ± 3.40^a^ (*N* = 650 eggs)	54.50 ± 3.20^b^ (*N* = 520 eggs)	46.22 ± 3.10^b^ ^c^ (*N* = 380 eggs)	77.80 ± 3.15^a^ (*N* = 660 eggs)	50.88 ± 3.30^b^ ^c^ (*N* = 340 eggs)	22.40 ± 2.80^d^ (*N* = 120 eggs)
**Newly hatched nymphs**	28.09 ± 3.96^a^ (*N* = 19)	21.50 ± 3.55^a^ (*N* = 20)	16.51 ± 3.20^b^ (*N* = 13)	12.65 ± 2.10^b^ ^c^ (*N* = 9)	27.20 ± 3.85^a^ (*N* = 19)	16.10 ± 2.80^b^ ^c^ (*N* = 10)	4.10 ± 0.95^d^ (*N* = 4)
**Mean nymph weight (mg)**	1.38 ± 0.03^a^ (*N* = 160)	1.39 ± 0.04^a^ (*N* = 180)	1.33 ± 0.04^a^ ^b^ (*N* = 120)	1.24 ± 0.04^b^ ^c^ (*N* = 85)	1.37 ± 0.03^a^ (*N* = 160)	1.25 ± 0.04^b^ ^c^ (*N* = 95)	1.10 ± 0.05^c^ (*N* = 40)

Note: Within each inbreeding treatment (BS or MS), means followed by the same letter (a, b, c, ab, bc) are not significantly different (*P* > 0.05); means followed by different letters are significantly different (*P* < 0.05). Letters compare generations within the same treatment only.

#### Second-Clutch Hatching Success Rate

Hatching rate was consistently reduced in the second clutch compared with the first clutch across all treatments (Table 3). Outbred controls showed 79.35% hatching success in the second clutch. Within the BS regime, hatching success declined significantly from 64.20% (F_1_) to 54.50% (F_2_) and 46.22% (F_3_) (F(2,57) = 11.20, *P* < 0.001). Within the MS regime, hatching success declined from 77.80% (F_1_) to 50.88% (F_2_) and 22.40% (F_3_) (*F*(2,57) = 20.33, *P* < 0.001), with MS F_3_ representing a 71.8% reduction compared with controls.

#### Second-Clutch Newly Hatched Nymph Count

The number of newly hatched nymphs from the second clutch showed the most severe declines of all reproductive parameters (Table 3). Outbred controls produced 28.09 ± 3.96 nymphs. Within the BS regime, nymph counts declined from 21.50 (F_1_) to 16.51 (F_2_) and 12.65 (F_3_) (*F*(2,57) = 15.53, *P* < 0.001). Within the MS regime, nymph counts declined from 27.20 (F_1_) to 16.10 (F_2_) and 4.10 (F_3_) (*F*(2,57) = 26.42, *P* < 0.001), with MS F_3_ representing an 85.4% reduction compared with controls.

#### Second-Clutch Mean Nymph Weight

Mean nymph weight from the second clutch showed a transient increase in BS F_1_ (1.39 ± 0.04 mg) compared with controls (1.38 ± 0.03 mg), before declining in subsequent generations within the BS regime (Table 3). Within the BS regime, mean nymph weight declined from 1.39 ± 0.04 mg (F_1_) to 1.33 ± 0.04 mg (F_2_) and 1.24 ± 0.04 mg (F_3_) (*F*(2,57) = 5.21, *P* = 0.008). Within the MS regime, mean nymph weight declined from 1.37 ± 0.03 mg (F_1_) to 1.25 ± 0.04 mg (F_2_) and 1.10 ± 0.05 mg (F_3_) (*F*(2,57) = 6.78, *P* = 0.002), with MS F_3_ representing a 20.3% reduction compared with controls.

### Lifetime Reproductive Parameters

#### Likelihood of Producing a Second Clutch

The likelihood of producing a second clutch showed an initial increase in BS F_1_ (84.20%) compared with controls (82.61%), but declined rapidly thereafter within the BS regime. Within the BS regime, the likelihood declined significantly from 84.20% (F_1_) to 65.73% (F_2_) and 44.50% (F_3_) (*F*(2,57) = 9.90, *P* < 0.001). Within the MS regime, the likelihood declined from 81.50% (F_1_) to 50.43% (F_2_) and 18.25% (F_3_) (*F*(2,57) = 15.92, *P* < 0.001), with MS F_3_ representing a 77.9% reduction compared with controls (Table 4).

**Table 4.** Lifetime Reproductive Parameters Across Generations (Mean ± SE)

Parameter	Control	BS F_1_	BS F_2_	BS F_3_	MS F_1_	MS F_2_	MS F_3_
**Likelihood of second clutch (%)**	82.61^a^ (*N* = 20)	84.20^a^ (*N* = 20)	65.73^b^ (*N* = 20)	44.50^c^ (*N* = 20)	81.50^a^ (*N* = 20)	50.43^c^ (*N* = 20)	18.25^d^ (*N* = 20)
**Relative investment in second clutch**	31.16 ± 3.43^a^ (*N* = 19)	32.40 ± 3.30^a^ (*N* = 20)	26.65 ± 3.15^a^ ^b^ (*N* = 13)	19.40 ± 3.11^b^ ^c^ (*N* = 9)	30.80 ± 3.40^a^ (*N* = 19)	19.88 ± 2.40^b^ ^c^ (*N* = 10)	8.60 ± 1.40^d^ (*N* = 4)
**Lifetime egg number**	101.00 ± 4.87^a^ (*N* = 20)	99.80 ± 4.85^a^ (*N* = 20)	80.06 ± 4.38^b^ (*N* = 20)	60.72 ± 3.95^c^ (*N* = 20)	100.20 ± 4.90^a^ (*N* = 20)	70.05 ± 3.68^b^ ^c^ (*N* = 20)	39.10 ± 2.50^d^ (*N* = 20)
**Lifetime newly hatched nymphs**	83.44 ± 4.10^a^ (*N* = 20)	77.20 ± 4.20^a^ (*N* = 20)	56.57 ± 3.68^b^ (*N* = 20)	40.75 ± 3.02^c^ (*N* = 20)	82.10 ± 4.15^a^ (*N* = 20)	50.97 ± 3.40^b^ ^c^ (*N* = 20)	18.50 ± 1.90^d^ (*N* = 20)

Note: Within each inbreeding treatment (BS or MS), means followed by the same letter (a, b, c, ab, bc) are not significantly different (*P* > 0.05); means followed by different letters are significantly different (*P* < 0.05). Letters compare generations within the same treatment only.

#### Relative Investment in the Second Clutch

Relative investment in the second clutch declined across generations within each inbreeding regime. Within the BS regime, relative investment declined from 32.40 (F_1_) to 26.65 (F_2_) and 19.40 (F_3_) (*F*(2,57) = 11.20, *P* < 0.001). Within the MS regime, relative investment declined from 30.80 (F_1_) to 19.88 (F_2_) and 8.60 (F_3_) (*F*(2,57) = 14.87, *P* < 0.001), with MS F_3_ representing a 72.4% reduction compared with controls (Table 4).

#### Lifetime Egg Number

Lifetime egg number declined progressively across generations within each inbreeding regime. Outbred controls produced 101.00 eggs. Within the BS regime, lifetime egg number declined significantly from 99.80 (F_1_) to 80.06 (F_2_) and 60.72 (F_3_) (*F*(2,57) = 14.90, *P* < 0.001). Within the MS regime, lifetime egg number declined from 100.20 (F_1_) to 70.05 (F_2_) and 39.10 (F_3_) (*F*(2,57) = 17.92, *P* < 0.001), with MS F_3_ representing a 61.3% reduction compared with controls (Table 4).

#### Lifetime Newly Hatched Nymphs

Lifetime newly hatched nymphs showed the starkest collapse of all reproductive parameters (Table 4). Outbred controls produced 83.44 nymphs. Within the BS regime, lifetime nymphs declined from 77.20 (F_1_) to 56.57 (F_2_) and 40.75 (F_3_) (*F*(2,57) = 16.75, *P* < 0.001). Within the MS regime, lifetime nymphs declined from 82.10 (F_1_) to 50.97 (F_2_) and 18.50 (F_3_) (*F*(2,57) = 21.65, *P* < 0.001), with MS F_3_ representing a 77.8% reduction compared with controls.

### Sex-Specific Survival Parameters

#### Female Survival

Female survival dropped progressively across generations within each inbreeding regime (Fig. 1). Under BS mating, female survival declined significantly from 84.2% (control) to 56.8% (F_1_), 41.95% (F_2_), and 29.2% (F_3_) (*F*(2,57) = 29.40, *P* < 0.001), representing a 65.3% reduction by F_3_. Under MS backcrossing, female survival declined from 83.5% (F_1_) to 54.6% (F_2_) and 25.0% (F_3_) (*F*(2,57) = 3.34, *P* = 0.042), representing a 70.3% reduction by F_3_.

**Fig. 1.**
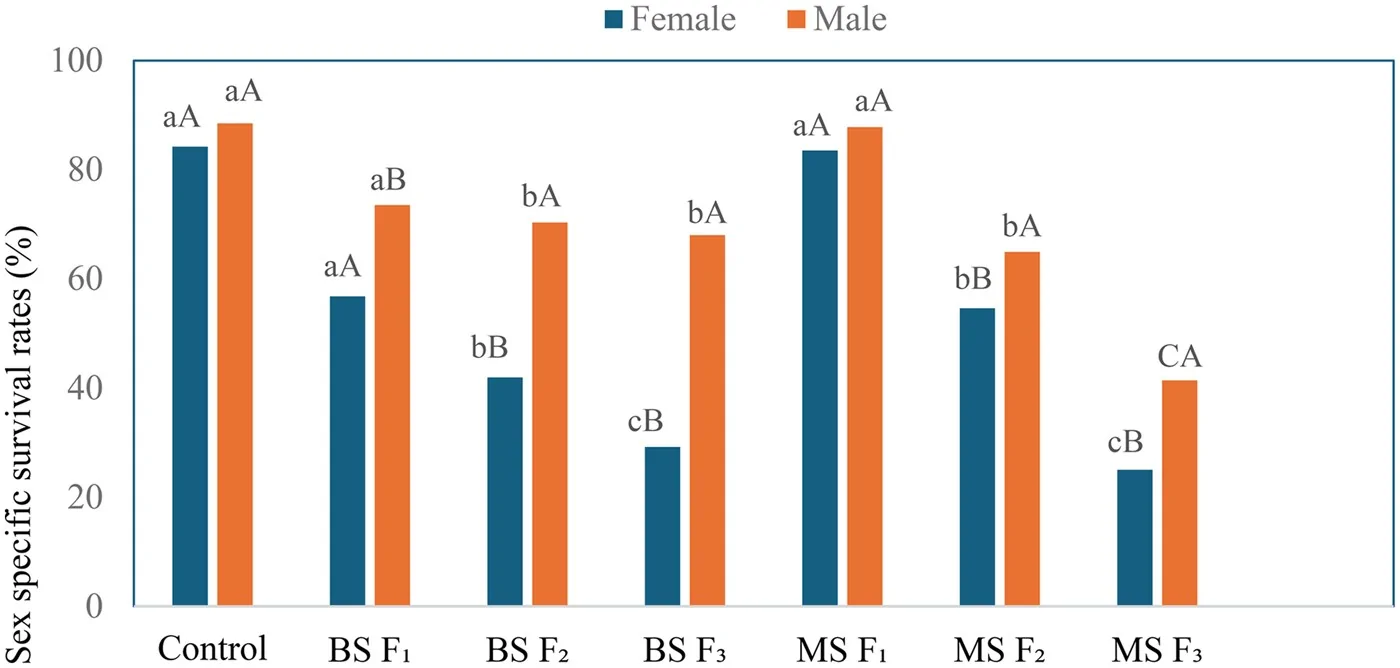
Sex specific survival rates (%) across generations under sibling (BS) and mother-son (MS) inbreeding regimes. Uppercase letters (A, B, C): between sexes within the same treatment-generation combination. Lowercase letters (a, b, c): indicate significant differences among treatment-generation combinations within the same sex.

#### Male Survival

Under BS mating, male survival dropped from 88.5% (control) to 73.5% (F_1_) but then stabilized at 70.3% (F_2_) and 68.0% (F_3_), representing only a 23.2% reduction by F_3_ (*F*(2,57) = 3.12, *P* = 0.051) **(**Fig. 1**)**. Under MS backcrossing, male survival continued its decline from 87.8% (F_1_) to 64.95% (F_2_) and crashed to 41.4% (F_3_) (*F*(2,57) = 12.08, *P* < 0.001), representing a 53.2% reduction by F_3_. The Treatment × Sex interaction (*P* = 0.013) and Treatment × Sex × Generation interaction (*P* = 0.011) were statistically significant, supporting the divergence in sex-specific survival curves under the 2 regimes.

### Sex-Specific Morphological Parameters

#### Overall Malformation Rate

Phenotypic malformations were extremely rare in outbred controls, occurring at a rate of less than 0.5%. Within the BS regime, the overall malformation rate increased from 3.2% (F_1_) to 9.8% (F_2_) and 14.8% (F_3_), with the majority (78.4%) occurring in females. Within the MS regime, the overall malformation rate increased from <0.5% (F_1_) to 12.4% (F_2_) and 32.2% (F_3_), with nearly equal distribution between sexes.

#### Female-Specific and Male-Specific Malformation Rates

Female malformation rates increased progressively across generations within each inbreeding regime (Table 5). Within the BS regime, female malformation rates increased from 3.2% (F_1_) to 9.8% (F_2_) and 11.6% (F_3_) (*F*(2,57) = 12.25, *P* < 0.001). Female malformations were primarily wing distortions (72.3% of female malformations). Within the MS regime, female malformation rates increased from <0.5% (F_1_) to 12.4% (F_2_) and 16.2% (F_3_) (*F*(2,57) = 24.06, *P* < 0.001).

**Table 5.** Sex-specific malformation rates (%) across generations

Sex	Control	BS F_1_	BS F_2_	BS F_3_	MS F_1_	MS F_2_	MS F_3_
**Female**	<0.5^a^ (*N* = 1,200)	3.2^a^ (*N* = 1,150)	9.8^b^ (*N* = 980)	11.6^b^ (*N* = 850)	<0.5^a^ (*N* = 1,180)	12.4^b^ (*N* = 820)	16.2^c^ (*N* = 620)
**Male**	<0.5^a^ (*N* = 1,100)	1.1^a^ (*N* = 1,050)	3.1^b^ (*N* = 920)	3.2^b^ (*N* = 780)	<0.5^a^ (*N* = 1,080)	10.8^b^ (*N* = 760)	16.0^c^ (*N* = 580)
**Overall**	<0.5^a^ (*N* = 2,300)	3.2^a^ (*N* = 2,200)	9.8^b^ (*N* = 1,900)	14.8^c^ (*N* = 1,630)	<0.5^a^ (*N* = 2,260)	12.4^b^ (*N* = 1,580)	32.2^d^ (*N* = 1,200)

Note: Within each inbreeding treatment (BS or MS), means followed by the same letter (a, b, c, ab, bc) are not significantly different (*P* > 0.05); means followed by different letters are significantly different (*P* < 0.05). Letters compare generations within the same treatment only.

#### Male Malformation Rates Remained Low Within the Bs Regime

1.1% (F_1_), 3.1% (F_2_), and 3.2% (F_3_) (F(2,57) = 1.93, *P* = 0.154). Male malformations under BS were mostly minor tergite abnormalities (21.6% of total malformations). Within the MS regime, male malformation rates increased from <0.5% (F_1_) to 10.8% (F_2_) and 16.0% (F_3_) (*F*(2,57) = 21.33, *P* < 0.001), with severe malformations including tergite fusion, antennal fusion, and compound wing distortions observed in MS males. The inbreeding Type × Sex interaction was highly significant for the binomial logistic model (*P* < 0.001) (Table 5).

### Fluctuating Asymmetry (FA) Parameters

#### Female Fluctuating Asymmetry (FA)

Female wing FA increased significantly across generations within each inbreeding regime (Table 6). Control values were 0.012 mm. Within the BS regime, female FA increased from 0.015 mm (F_1_) to 0.040 mm (F_2_) and 0.062 mm (F_3_) (*F*(2,57) = 28.60, *P* < 0.001). Within the MS regime, female FA increased from 0.013 mm (F_1_) to 0.048 mm (F_2_) and 0.068 mm (F_3_) (*F*(2,57) = 31.14, *P* < 0.001).

**Table 6.** Sex-specific fluctuating asymmetry (FA, mm) across generations (mean ± SE)

Sex	Control	BS F_1_	BS F_2_	BS F_3_	MS F_1_	MS F_2_	MS F_3_
**Female**	0.012 ± 0.002^a^ (*N* = 50)	0.015 ± 0.003^a^ (*N* = 100)	0.040 ± 0.004^b^ (*N* = 50)	0.062 ± 0.005^c^ (*N* = 50)	0.013 ± 0.002^a^ (*N* = 100)	0.048 ± 0.004^b^ (*N* = 50)	0.068 ± 0.006^c^ (*N* = 25)
**Male**	0.011 ± 0.002^a^ (*N* = 50)	0.011 ± 0.002^a^ (*N* = 100)	0.013 ± 0.002^a^ (*N* = 50)	0.014 ± 0.002^a^ (*N* = 50)	0.012 ± 0.002^a^ (*N* = 100)	0.026 ± 0.003^b^ (*N* = 50)	0.038 ± 0.004^c^ (*N* = 25)

Note: Within each inbreeding treatment (BS or MS), means followed by the same letter (a, b, c, ab, bc) are not significantly different (*P* > 0.05); means followed by different letters are significantly different (*P* < 0.05). Letters compare generations within the same treatment only.

#### Male Fluctuating Asymmetry (Fa)

Male wing FA within the BS regime did not change significantly across generations: control values were 0.011 mm, BS F_1_ showed 0.011 mm, BS F_2_ showed 0.013 mm, and BS F_3_ showed 0.014 mm (*F*(2,57) = 0.86, *P* = 0.420). Within the MS regime, male FA increased from 0.012 mm (F_1_) to 0.026 mm (F_2_) and 0.038 mm (F_3_) (*F*(2,57) = 16.20, *P* < 0.001). For the specific two-group comparison of BS F_3_ vs. MS F_3_ male FA, a *t*-test was used (*t*_7__3_ = 5.67, *P* < 0.001) (Table 6).

### Sex Ratio Data

#### Offspring Sex Ratio

The proportion of males remained near the expected 1:1 ratio in the Control and in the outbred F_1_ generation of both inbreeding regimes (0.51–0.52) (Fig. 2). Within the BS regime, the proportion of males increased significantly from 0.51 ± 0.03 (F_1_) to 0.58 ± 0.04 (F_2_) and 0.61 ± 0.04 (F_3_) (*F*(2,57) = 6.05, *P* = 0.004). Within the MS regime, the male bias was stronger and emerged earlier, rising from 0.52 ± 0.03 (F_1_) to 0.62 ± 0.04 (F_2_) and 0.71 ± 0.04 (F_3_) (*F*(2,57) = 14.70, *P* < 0.001).

**Fig. 2.**
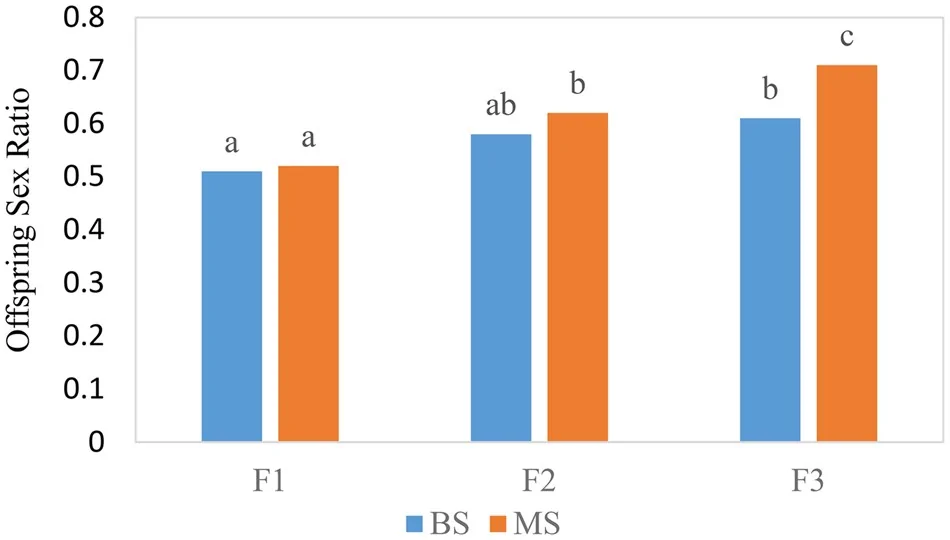
Offspring sex ratio (proportion of males) across generations under sibling (BS) and mother-son (MS) inbreeding regimes. Different letters indicate significant difference within the same treatment-generation and Similar letters indicate non significant difference within the same treatment-generation.

### Population Growth Parameters

#### Net Reproductive Rate (*R*_0_)

The net reproductive rate collapsed progressively across generations within each inbreeding regime. Control values were 48.52. Within the BS regime, *R*_0_ declined from 33.50 (F_1_) to 23.28 (F_2_) and 14.15 (F_3_) (*F*(2,57) = 38.90, *P* < 0.001). Within the MS regime, *R*_0_ declined from 47.80 (F_1_) to 19.70 (F_2_) and 4.85 (F_3_) (*F*(2,57) = 68.32, *P* < 0.001), with MS F_3_ representing a 90.0% reduction compared with controls (Table 7).

**Table 7.** Population Growth Parameters Across Generations (Mean ± SE)

Parameter	Control	BS F_1_	BS F_2_	BS F_3_	MS F_1_	MS F_2_	MS F_3_
***R*_0_**	48.52 ± 3.12^a^ (*N* = 20)	33.50 ± 2.90^a^ (*N* = 20)	23.28 ± 2.20^b^ (*N* = 20)	14.15 ± 1.60^c^ (*N* = 20)	47.80 ± 3.10^a^ (*N* = 20)	19.70 ± 1.95^b^ (*N* = 20)	4.85 ± 0.90^d^ (*N* = 20)
***r* (d^−^^1^)**	0.185 ± 0.011^a^ (*N* = 20)	0.145 ± 0.010^a^ (*N* = 20)	0.115 ± 0.009^b^ (*N* = 20)	0.088 ± 0.008^c^ (*N* = 20)	0.183 ± 0.011^a^ (*N* = 20)	0.104 ± 0.009^b^ (*N* = 20)	0.024 ± 0.005^d^ (*N* = 20)
**Λ**	1.203 ± 0.013^a^ (*N* = 20)	1.156 ± 0.012^a^ (*N* = 20)	1.122 ± 0.011^b^ (*N* = 20)	1.092 ± 0.010^c^ (*N* = 20)	1.201 ± 0.013^a^ (*N* = 20)	1.110 ± 0.011^b^ (*N* = 20)	1.024 ± 0.006^d^ (*N* = 20)
***T* (days)**	21.05 ± 0.85^a^ (*N* = 20)	23.50 ± 0.90^a^ (*N* = 20)	27.31 ± 0.98^b^ (*N* = 20)	30.12 ± 1.05^c^ (*N* = 20)	21.20 ± 0.86^a^ (*N* = 20)	28.15 ± 1.00^b^ (*N* = 20)	34.60 ± 1.15^d^ (*N* = 20)

Note: Within each inbreeding treatment (BS or MS), means followed by the same letter (a, b, c, ab, bc) are not significantly different (*P* > 0.05); means followed by different letters are significantly different (*P* < 0.05). Letters compare generations within the same treatment only.

#### Intrinsic Rate of Increase (*r*, d^−^^1^)

The intrinsic rate of increase declined progressively across generations within each inbreeding regime (Table 7). Control values were 0.185 ± 0.011 d^−^^1^. Within the BS regime, *r* declined from 0.145 d^−^^1^ (F_1_) to 0.115 d^−^^1^ (F_2_) and 0.088 d^−^^1^ (F_3_) (*F*(2,57) = 35.40, *P* < 0.001). Within the MS regime, *r* declined from 0.183 d^−^^1^ (F_1_) to 0.104 d^−^^1^ (F_2_) and 0.024 d^−^^1^ (F_3_) (*F*(2,57) = 62.15, *P* < 0.001), with MS F_3_ representing an 87.0% reduction compared with controls. Critically, the *r* value of 0.024 d^−^^1^ indicates that MS F_3_ populations were barely maintaining themselves, with growth approaching zero.

#### Finite Rate of Increase (*λ*)

The finite rate of increase declined progressively across generations within each inbreeding regime (Table 7). Control values were 1.203. Within the BS regime, *λ* declined from 1.156 (F_1_) to 1.122 (F_2_) and 1.092 (F_3_) (*F*(2,57) = 32.10, *P* < 0.001). Within the MS regime, *λ* declined from 1.201 (F_1_) to 1.110 (F_2_) and 1.024 (F_3_) (*F*(2,57) = 58.40, *P* < 0.001), with MS F_3_ indicating that populations were growing by only 2.4% per day, compared with 20.3% per day in controls.

#### Mean Generation Time (*T*, Days)

Generation time extended progressively under inbreeding within each regime (Table 7). Control values were 21.05 d. Within the BS regime, *T* increased from 23.50 d (F_1_) to 27.31 d (F_2_) and 30.12 d (F_3_) (*F*(2,57) = 26.80, *P* < 0.001). Within the MS regime, *T* increased from 21.20 d (F_1_) to 28.15 d (F_2_) and 34.60 d (F_3_) (*F*(2,57) = 49.10, *P* < 0.001), with MS F_3_ representing a 64.3% increase in generation time compared with controls.

## Discussion

In both inbreeding systems, there was a progressive decline in the number of eggs in the first clutch from F_1_ to F_3_ generations, with MS lines having a more pronounced decline (65.20 to 34.20 eggs) than BS lines (64.85 to 48.12 eggs) (Table 2). Such a decrease indicates that inbreeding negatively affects oogenesis and oviposition ability; this may be related to the growing homozygosity of the loci involved in the development of ­ovarian structures (Roff 1997, Lynch and Walsh 1998). The decreasing success rate of the hatching of the eggs in the first clutch (from 83.95% for control to 58.40% for BS F_3_ and 41.15% for MS F_3_) is another proof of egg quality deterioration (Saito et al. 2000). In this case, the survival rate of newborn nymphs in the first clutch was decreased by about 75% in MS F_3_ compared to the control (14.05 vs. 55.35), showing that the result of low egg count and low hatchability leads to very weak early stage recruitment of population (Henter 2003). Transient increase in the mean nymph weight found in BS F_1_ (1.46 mg vs. control 1.45 mg) can be especially considered as an important heterosis effect, as it shows that the masking of the deleterious alleles in the first outbred derived generations is characteristic of heterosis (Charlesworth and Charlesworth 1987, Keller and Waller 2002). But in F_3_, the mean nymph weight was already lowered to 1.31 mg (BS) and 1.18 mg (MS), as it shows that when homozygosity increased, the masking effect turned into recessive allele expression (Haag and Ebert 2004). The distinct pattern observed in Table 2 for MS F_1_ (egg number 65.20, newly hatched nymphs 53.80) compared to Control and BS F_1_ is explained by the fact that MS F_1_ is outbred, produced by mating an F_0_ virgin female to an unrelated F_0_ male from the outbred stock colony. MS F_1_ is therefore genetically equivalent to BS F_1_ and the Control, as all three are produced by outbred parents.

### Second-Clutch Reproductive Parameters (Hatching Success and Newly Hatched Nymphs)

The degree of deterioration was always worse in the second clutch (days 6–10) than in the first clutch for all groups, demonstrating that the females were experiencing considerable physiological exhaustion. The hatching rate in the second clutch decreased from 79.35% (control group) to 46.22% (BS F_3_) and a dramatic 22.40% (MS F_3_) (Table 3). Such a decrease was statistically significant (*P* < 0.01) compared with the simultaneous reduction of the hatching rate in the first clutch (paired *t*-test). This shows that inbred females do not have the physiological ability to maintain oocyte quality during prolonged oviposition periods (Mousseau and Fox 1998, Müller and Müller 2016). The above phenomenon is congruent with the interference in vitellogenesis and oocyte provisioning in response to increased genetic load, as has been noted in haplodiploid mites in which the deleterious genes carried by females mainly influence fecundity (Bates et al. 2015, Saito et al. 2000, Saito & Mori 2000). It must be mentioned that the fall in the number of second clutch nymphs that have just hatched, from 28.09 (control) to 4.10 (MS F_3_), amounts to an 85.4% reduction, indicating that inbreeding depression has its maximum impact when females reach the late reproductive stage (Southwood and Henderson 2000). The above phenomenon carries significance in terms of population persistence because successful reproduction for a long period of time is important for population growth (Chi 1988, Müller and Müller 2016).

The second-clutch egg number declined from 35.42 (control) to 17.85 (BS F_3_) and 6.45 (MS F_3_), representing a 49.6% and 81.8% reduction, respectively. This decline was steeper than the first-clutch decline (27.7% for BS F_3_ and 48.6% for MS F_3_), indicating that inbred females experience greater physiological exhaustion during later reproductive bouts. The second-clutch mean nymph weight also declined significantly, from 1.38 mg (control) to 1.24 mg (BS F_3_) and 1.10 mg (MS F_3_), representing a 10.1% and 20.3% reduction, respectively. The transient heterotic increase in BS F_1_ (1.39 mg) compared to controls (1.38 mg) is consistent with the masking of deleterious alleles in the first outbred-derived generation. The subsequent decline in BS F_2_ and BS F_3_ reflects the increasing homozygosity and expression of recessive deleterious alleles.

### Lifetime Reproductive Parameters (Likelihood of Second Clutch, Relative Investment, Lifetime Egg Number, and Lifetime Nymphs)

The lifetime statistics demonstrate the cumulative effects of inbreeding on fitness over multiple generations. There was an unusual trend in the probability of producing another clutch, where the probability increased slightly in BS F_1_ (84.20% compared to 82.61% in controls), once again indicating temporary heterosis (Charlesworth and Charlesworth 1987), but declined drastically to 44.50% in BS F_3_ and finally to 18.25% in MS F_3_. This dramatic decline clearly shows that inbred females are unable to reproduce for long periods of time physiologically (Henter 2003, Tien et al. 2015). Relative investment in the second clutch (output of second clutch/first clutch output) decreased from 31.16 (control) to merely 8.60 in MS F_3_, indicating that inbred females invest relatively less in their subsequent broods (Roff 1997, Mousseau and Fox 1998). Total egg production in lifetime dropped from 101.00 (control) to 39.10 in MS F_3_ (61.3% reduction), whereas total new nymphs in lifetime dropped from 83.44 to 18.50 (77.8% reduction). It is important to note here that the decrease in total nymphs in lifetime is much more pronounced than the drop in total eggs in lifetime. This observation indicates that inbreeding decreases not only the oviposition rate but also the egg viability and larval survival rates (Woldemelak 2024). These results are consistent with the prediction that purging does not take place fast for the mutations that affect the specific reproductive features of females, and such mutations will accumulate in haplodiploids as they are protected in males (Hedrick 1994, Henter 2003).

### Sex-Specific Survival Parameters (Female and Male Survival Rates)

The data on survival (Table 4) constitute the strongest proof of the haploid purging hypothesis and the failure of this phenomenon with backcross mating (Henter 2003, Tien et al. 2015). Female survival rate decreased in a progressive fashion from 84.2% (control) to 29.2% (F_3_)—a 65.3% reduction. In case of males, survival rate decreased from 88.5% to 73.5% (F_1_) but remained relatively constant from F_1_ onwards (68.0% (F_3_)), resulting in only 23.2% decrease. The reason for such stability lies in haploid purging: the deleterious recessive alleles are immediately exposed to selection in haploid males, and the surviving males have a purged genome, hence providing protection to male fitness against inbreeding over multiple generations (Godfray 1994, Henter 2003).

Under the MS backcrossing regime, however, this protective effect was totally lost. While male survival dropped to 41.4% at F_3_ (a 53.2% decrease), the drop for females was even more extreme, down to only 25.0% (a 70.3% decrease). Treatment × Sex interaction (*P* = 0.013) and Treatment × Sex × Generation interaction (*P* = 0.011) statistically support the divergence in sex-specific survival curves under the 2 different regimes. Under the MS regime, a son becomes obliged to pass 100% of his maternal genes back to his mother; if the latter harbors the deleterious mutations restricted in females and not expressed in males, then the instant backcrossing will cause exposure of those mutations in the next generation, thereby losing the sex bias (Saito et al. 2000, Saito and Mori 2000). Such results confirm theoretical expectations about the strong influence of the mating regime and the sex specificity of the deleterious mutations on the efficiency of purging (Hedrick 1994).

### Morphological Parameters

This is confirmed visually and quantitatively through the morphological results (Tables 5 and 6). Outbred controls showed no deformities (less than 0.5%), which indicates high developmental stability. For BS, the deformity ratio was 14.8% at the F_3_ generation, with a highly skewed female proportion (78.4% of malformations were seen in females, wing deformities being common). The predominance of females is related to the presence of recessive alleles which are masked in males and unmasked in diploid females (Godfray 1994, Chapman 1998). In a striking contrast, the MS F_3_ showed a 32.2% deformity ratio with equal distribution by sex (50.3% female, 49.7% male), including tergite and antenna fusions. The interaction of Inbreeding Type and Sex was highly significant for the binomial logistic model (*P* < 0.001), implying that there were substantial differences in sex bias between the 2 regimes. Equal sex distribution of individuals in the MS regime implied that the process of backcrossing led to mutations in developmental genes important to both sexes, rather than sex specific characteristics (Saito et al. 2000). Fluctuating asymmetry (FA), which is an important measure of developmental instability (Palmer and Strobeck 1986), exhibited similar trends. FA of wings of females was five times higher at F_3_ (0.062 mm vs. 0.012 mm in control; *F*(2,57) = 31.14, *P* < 0.001), while male FA at BS was constant (0.014 mm, *P* = 0.42). At the same time, male FA in the MS regime increased significantly to 0.038 mm at F_3_ (*t*_7__3_ = 5.67, *P* < 0.001), implying that backcrossing broke the developmental stability of males, which had been preserved by sibling mating (Palmer and Strobeck 1986, Slatkin 1987). The results support the idea that fluctuating asymmetry is the result of the accumulated effects of genetic stress, but the male haploid genome serves as protection from genetic stress only when the purging process can take place (Palmer and Strobeck 1986, Henter 2003).

### Population Demographic Parameters (Net Reproductive Rate *R*_0_, Intrinsic Rate r, Finite Rate *λ*, and Mean Generation Time *T* )

The demographic traits (Table 7) offer the ultimate indicators of population-level fitness and invasiveness (Chi 1988, Müller and Müller 2016). The net reproductive rate (*R*_0_), defined as the average number of female offspring produced by each female, declined from 48.52 (controls) to 14.15 (BS F_3_) and dramatically to 4.85 (MS F_3_), a decline of 90.0%. *R*_0_ value less than 5 shows that each female was producing fewer than 5 daughters, which is too few females for a population to survive under natural mortality (Chi 1988). The intrinsic rate of increase (*r*), which is the most important indicator of a population growth capacity, dropped from 0.185 d^−^^1^ (controls) to 0.088 d^−^^1^ (BS F_3_) and 0.024 d^−^^1^ (MS F_3_). The MS F_3_ rate is 87.0% lower than the controls and suggests that the population was growing at a rate of 13% of the controls, not much better than replacement levels (Southwood and Henderson 2000, Müller and Müller 2016).

In parallel, the rate of finite increase (*λ*) decreased from 1.203 to 1.024 in MS F_3_ populations, and hence the population reproduced at an average rate of 2.4% a day compared to the 20.3% growth in control populations. A low *λ* value shows that any extra stress in the environment, such as the presence of pesticides or predation, will cause the population to decline (Bielza 2008, Otto and Gerstein 2008). The mean generation time (*T*) increased from 21.05 d (control) to 34.60 d in MS F_3_; this is an increase of 64.3%. This large increase shows that inbred populations will take much more time to complete one generation, and therefore will have difficulty recovering from disturbance, adapting to new environments, or evolving resistance (Gao et al. 2012). Overall, demographic parameters show that MS backcrossing will drive the population to the point of extinction within three generations (Kirk 2002, Reitz et al. 2020).

### Implications for Integrated Pest Management and Future Directions

The findings imply important considerations for the management of *F. occidentalis* (Pergande, 1895) (Kirk 2002, Reitz et al. 2020). Fragmentation of populations in greenhouses and agricultural systems leads to low gene flow among subpopulations, making inbreeding inevitable (Funderburk et al. 2000, Morse and Hoddle 2006). The study reveals that inbreeding over 3 generations makes the population vulnerable to demographic collapse, which implies that inbreeding alone can prevent survival without additional measures (Caughley 1994). Low values of *r* and *λ* under inbreeding make evolution of resistance much slower, as rare resistance alleles cannot be selected fast enough (Bielza 2008). Additionally, inbred populations with a high percentage of malformations (32.2%) and long generation time would be much more vulnerable to biocontrol agents, including predatory mites and entomopathogenic fungi (Kirk 2002, Reitz et al. 2020).

Further research must focus on transcriptomic sequencing to find the gene networks that are specifically responsible for these asymmetric fitness costs (Saito et al. 2000, Saito and Mori 2000), while field investigations must confirm these findings within the context of natural populations (Funderburk et al. 2000, Morse and Hoddle 2006). Knowledge of the genetic and population-level implications of inbreeding in haplodiploid pests will allow us to design better IPM programs (Stern et al. 1959, Kogan 1998).
